# Genome-wide analysis of canine oral malignant melanoma metastasis-associated gene expression

**DOI:** 10.1038/s41598-019-42839-x

**Published:** 2019-04-24

**Authors:** K. L. Bowlt Blacklock, Z. Birand, L. E. Selmic, P. Nelissen, S. Murphy, L. Blackwood, J. Bass, J. McKay, R. Fox, S. Beaver, M. Starkey

**Affiliations:** 1 Animal Health Trust, Newmarket, Suffolk, UK; 2 Department of Veterinary Clinical Sciences, The Ohio State University, Columbus, Ohio USA; 3 Dick White Referrals, Newmarket, Suffolk, UK; 4 Institute of Veterinary Science, University of Liverpool, Liverpool, UK; 5 IDEXX Laboratories, Ltd, Wetherby, UK; 6 Finn Pathologists, Harleston, UK; 7 Nationwide Laboratory Services, Poulton-le-Fylde, UK; 8 Present Address: The Royal (Dick) School of Veterinary Studies, University of Edinburgh, Edinburgh, UK; 9 Present Address: Finn Pathologists, Harleston, UK

**Keywords:** Cancer genomics, Cancer genomics

## Abstract

Oral malignant melanoma (OMM) is the most common canine melanocytic neoplasm. Overlap between the somatic mutation profiles of canine OMM and human mucosal melanomas suggest a shared UV-independent molecular aetiology. In common with human mucosal melanomas, most canine OMM metastasise. There is no reliable means of predicting canine OMM metastasis, and systemic therapies for metastatic disease are largely palliative. Herein, we employed exon microarrays for comparative expression profiling of FFPE biopsies of 18 primary canine OMM that metastasised and 10 primary OMM that did not metastasise. Genes displaying metastasis-associated expression may be targets for anti-metastasis treatments, and biomarkers of OMM metastasis. Reduced expression of *CXCL12* in the metastasising OMMs implies that the *CXCR4*/*CXCL12* axis may be involved in OMM metastasis. Increased expression of *APOBEC3A* in the metastasising OMMs may indicate *APOBEC3A*-induced double-strand DNA breaks and pro-metastatic hypermutation. DNA double strand breakage triggers the DNA damage response network and two Fanconi anaemia DNA repair pathway members showed elevated expression in the metastasising OMMs. Cross-validation was employed to test a Linear Discriminant Analysis classifier based upon the RT-qPCR-measured expression levels of *CXCL12*, *APOBEC3A* and *RPL29*. Classification accuracies of 94% (metastasising OMMs) and 86% (non-metastasising OMMs) were estimated.

## Introduction

Oral malignant melanomas (OMMs) are neoplastic proliferations of melanocytes, and are the most common oral tumour in dogs^1^. A predilection for OMM has been consistently suggested for Poodles^2,3^, and variously suggested for Golden Retrievers^2^, German Shepherd Dogs^4^ and Boxers^4^. OMM arise most frequently in the gingiva, but also develop in the buccal and labial mucosa, tongue and hard palate^5^. OMM is characterised by local invasion, recurrence after surgical resection, high metastatic propensity, and rapid progression from localised to advanced-stage disease^4,6^. Estimates of OMM metastasis range from 58–74% to regional lymph nodes, 14–67% to the lungs, and 65% to the tonsils^2,3,7^. Dogs with regional metastases treated with surgery and or radiotherapy have shorter survival times than dogs without metastases^8^. Adjunctive chemotherapy has not been shown to increase survival^9^, and data on the impact of the xenogeneic DNA vaccine^10–12^ for treatment of advanced OMM is equivocal. A median overall survival time of 335 days has been recently reported for OMM patients in receipt of a systemic adjuvant therapy following surgical excision of the primary tumour^9,13^.

Clinicopathological indicators of human cutaneous melanoma malignancy (tumour size and degree of pigmentation, presence of necrosis, ulceration or inflammation, rate of cell proliferation, and p53 expression level) have limited prognostic utility for canine melanoma, although mitotic index and Ki67 expression level appear to be of some value^14,15^. Canine OMM metastasis cannot be accurately predicted, nor is there an effective approach for early detection of metastasis. Cytologic or histologic examination of the mandibular and retropharyngeal lymph nodes is commonly performed during staging, and other lymph nodes may be selected for examination on the basis of lymphangiography. However, where OMM metastasis to regional lymph nodes occurs, it is not always detected by examination of the mandibular and retropharyngeal lymph nodes^16,17^.

Whilst OMM represents the most common type of melanoma in dogs^3,4,18^, oral mucosal melanoma (most often developing in the palate and gingiva^19^) accounts for only 1–8% of all human melanomas and around 0.5% of all human oral neoplasms^20,21^. However, human OMMs are also aggressive rapidly growing, invasive tumours that display metastatic rates of 66% (regional lymph nodes^22^), 53% (lung^23^), 36% (bone^23^), and 20% (liver and brain^23^), respectively. The 5-year survival rate is 15–25%^22,24^. Advanced human mucosal melanoma also has a low rate of response to adjuvant chemotherapy^25^. All human ethnic groups are affected by oral mucosal melanoma, although the Japanese appear to have an elevated susceptibility^26,27^.

Similarities between the somatic mutation profiles of human mucosal melanomas and canine OMMs suggests a possible overlapping molecular aetiology for UV-independent tumourigenesis. Putative activating mutations in Kit have been described in 7–16% of human mucosal melanomas^28,29^ and in 12% of canine OMM^30^, and mutations in NRAS have been reported in 3.9% of canine OMMs^3^ and in 10–22% of human mucosal melanomas^31–33^. Around 50% of human cutaneous melanomas have an activating BRAF mutation^34^, but BRAF mutations occur in only 4–9.5% of human mucosal melanomas^35,36^, and have not been found in canine OMMs^3^.

The metastatic cascade^37^ comprises a series of steps, which are believed to be at least partially mediated by the acquisition of metastasis-associated genetic and/or epigenetic alterations additional to those that drive tumour development^38^. These somatic changes may affect gene expression, and metastasis-associated gene expression signatures have been identified for many human tumours^39–41^. Transcriptional profiling has defined the stages in human cutaneous melanoma development and progression as a series of distinct ‘molecular events’, and implicated the involvement of sets of genes in the transition from primary to metastatic melanoma^42–44^. Gene expression signatures characteristic of human cutaneous melanoma metastases are detectable in primary cutaneous melanomas. A 1,864 gene expression signature derived from profiling cutaneous melanoma metastases was subsequently shown to delineate primary melanomas into two classes associated with significantly different relapse-free and overall survival^45^. Integration of functional and structural protein interaction data with primary and metastatic melanoma gene expression data enabled derivation of a panel of 6 genes which distinguished human primary and metastatic cutaneous melanoma and predicted melanoma-specific survival^46^. For human oral mucosal melanoma, comparative gene expression analysis of lymph node metastases and paired non-metastatic lymph nodes has elucidated the involvement of long non-coding RNAs in the regulation of metastasis-associated gene expression^47^. Metastasis-associated gene expression signatures identified in primary tumours may predict metastasis, and indeed assay of the expression of 15 genes is the basis of a routine test for human uveal melanoma metastasis^48^.

Gene expression profiling of canine cutaneous melanoma and melanocytoma has implicated the increased expression of genes involved in extracellular matrix-receptor interaction and the phosphoinositide 3-kinase/protein kinase B pathway in metastatic progression^49^, but no equivalent data is available for the more common canine OMM. Although we also recently identified pro-metastatic gene expression in an unrelated canine cancer (mast cell tumours^50^), the overall low degree of ‘overlap’ between the metastatic gene expression signatures identified for different human solid tumours^41,51^ affords the rationale for the gene expression profiling-based study of metastasis in different canine cancers, mirroring the approach adopted in the study of human tumour metastasis.

The high rate of mortality attributable to (conventional treatment-resistant) canine OMM metastasis is a significant welfare issue. It would be hugely beneficial for clinicians and owners alike to know whether a tumour was going to metastasise, while prevention of OMM metastasis would save the lives of most canine OMM patients. Establishing the role of dysregulated gene expression in canine OMM metastasis is an opportunity for identifying metastasis-associated biomarkers and possible anti-metastasis therapeutic targets.

In this project we performed comparative genome-wide expression profiling of archival biopsies of canine primary OMMs that metastasised and did not metastasise. We sought to identify metastasis-associated gene expression, and assess whether metastasising and non-metastasising OMMs could be delineated by the expression levels of genes associated with metastatic progression.

## Results

### Tumours subject to gene expression profiling

OMM biopsies from 42 dogs [29 bearing a metastasising (M) tumour and 13 bearing a non-metastasising (NM) tumour] qualified for the study. Through PowerAtlas^52^ analysis of human tumour gene expression datasets (from the Gene Expression Omnibus^53^), it was estimated that a ‘Discovery Rate’ of 73.1–81.7% at the 0.05 significance level would be afforded using 20 tumour samples in each of two ‘outcome groups’.

Several requests for FFPE OMM biopsies from patients fulfilling the NM inclusion criteria were made to all UK veterinary university teaching hospitals and three large multidisciplinary private practices in the UK, but no additional NM OMM biopsies could be recruited because of the regular use of xenogeneic vaccination for patients regardless of metastatic status at the time of presentation. One NM OMM biopsy was subsequently excluded because of a sub-optimal RNA concentration. The integrity of each OMM RNA sample was determined and 20 M OMM and 12 NM OMM sample groups, with similar RNA integrity ranges, assembled (Tables S1 and S2).

### Tumours included in differential expression analysis

Tumours with exon-level probe set expression profiles that differed from those of the majority of the 32 OMMs were identified by review of associated sample quality metrics^54^, and 2 M and 2 NM OMMs excluded (Tables S2 and S3). The gene-level probe set expression values in 18 M OMMs and 10 NM OMMs, respectively, were compared for 13,422 Transcript clusters (‘crosshyb_type’ = ‘1’; ‘category’ = ‘main’), for which the expression (above background) of ≥1 exon probe set was detected in at least 30% of the OMMs in the M and/or M groups.

The details of the dogs that bore the 18 M OMMs and 10 NM OMMs are presented in Table 1. Tumours in the M group were borne by 6 breeds and those in the NM group by 5 breeds. Tumours from Golden Retrievers and Labrador Retrievers, and cross bred dogs, were present in both groups. It is likely that the breed representation of the M and NM OMM groups reflect breed popularity and a predisposition to OMM development^2^; for example, OMMs from 4 Golden Retrievers were present in both the M and NM tumour groups. The median ages of the dogs with M and NM OMMs were comparable (10.85 and 10.25 years, respectively), whilst 61% and 60% of the M and NM OMM dogs were male or neutered male, respectively. No association between OMM gene expression profile and either gender, or age at diagnosis, could be gleaned by hierarchical clustering of the 28 OMMs according to the expression values of the 20% of Transcript Clusters (2,684) that had the highest variance in expression signal. Biopsies from 5 of the 8 Golden Retrievers were grouped in a single cluster (together with biopsies from a Dachshund and Bullmastiff), whilst the 3 remaining Golden Retriever OMM biopsies were partitioned in a second large sub-cluster with biopsies borne by 6 other breeds (Fig. S1). Although this suggests that further investigation is warranted to assess the impact of ‘genetic background’ on the somatic molecular profile of canine OMMs, from the perspective of identifying metastasis-associated gene expression it is important that the 8 Golden Retriever OMM biopsies were divided equally between the M and NM OMM groups. Although (after ‘outlier array’ exclusion) the mean age of a NM OMM FFPE biopsy was 1.6× years higher than the mean age of a M OMM FFPE biopsy there was no correlation between FFPE tumour biopsy age and tumour RNA integrity (Spearman rank correlation coefficient = 0.09, two-sided p-value: 0.61; Table S2).

**Table 1:** Dogs bearing oral malignant melanomas included in differential gene expression analysis.

Dog ID.	Breed	Sex	Age at diagnosis (Years)
**A**. **Metastasising OMMs**			
D1	Dachshund	FeN	3.5
LR1	Labrador Retriever	FeN	12.2
CB1	Cross breed	MaN	10.5
CB2	Cross breed	FeN	11.0
LR2	Labrador Retriever	Ma	11.6
D2	Dachshund	FeN	10.8
LR3	Labrador Retriever	FeN	11.1
GR1	Golden Retriever	FeN	10.9
CS1	Cocker Spaniel	Ma	10.0
GR2	Golden Retriever	MaN	9.5
CB3	Cross breed	FeN	7.8
CB4	Cross breed	MaN	10.9
CB5	Cross breed	MaN	8.0
GR3	Golden Retriever	MaN	10.0
GR4	Golden Retriever	Ma	12.4
BM1	Bullmastiff	MaN	10.0
CB6	Cross breed	Ma	12.0
BC1	Border Collie	MaN	12.0
		Mean and standard deviation	10.23 ± 2.12
		Median	10.85
		Interquartile range	1.48
**B**. **Non-metastasising OMMs**			
GD1	Great Dane	FeN	7.0
GR5	Golden Retriever	Ma	11.1
GR6	Golden Retriever	FeN	12.2
LR4	Labrador Retriever	Ma	11.3
LR5	Labrador Retriever	Ma	11.4
GR7	Golden Retriever	FeN	10.8
IT1	Irish Terrier	MaN	4.9
BF1	Bouvier des Flandres	MaN	7.6
GR8	Golden Retriever	FeN	7.0
CB7	Cross breed	MaN	9.7
		Mean and standard deviation	9.30 ± 2.48
		Median	10.25
		Interquartile range	4.10

Fe: Female; FeN: Neutered female; Ma: Male; MaN: Neutered male.

### Genes displaying differential expression in M and NM OMMs

**Figure 1:**
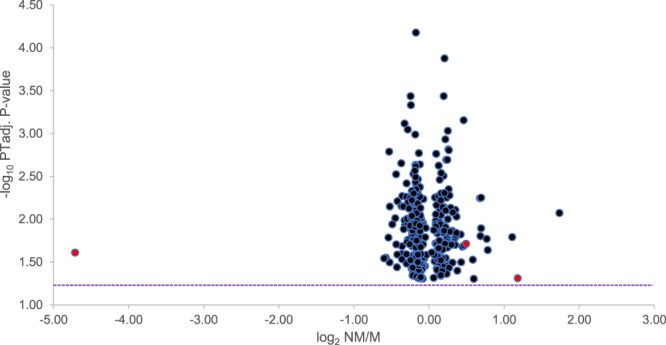
Genes differentially expressed between metastasising and non-metastasising OMMs. Exon microarray-measured expression of 331 genes in 18 metastasising (M) and 10 non-metastasising (NM) OMMs. The log_2_-transformed NM/M fold-change (x-axis) denotes the difference in gene expression between the M and NM OMMs. The minus log_10_-transformed permutation testing-adjusted t-test derived p-values (y-axis) indicates the statistical significances of gene expression differences. The dotted line illustrates the -log_10_ PTadj. p-value corresponding to a PTadj. p-value = 0.05. Red spheres represent the 3 genes subsequently employed in class prediction analysis.

In total, 331 Transcript clusters displayed a statistically significant difference in expression between M and NM OMMs. Of these, 191 exhibited increased expression in the M OMMs (Fig. 1). A >1.5-fold difference in expression between the M and NM OMMs was observed for 12 genes (Table 2). Significant sequence similarity to a mRNA encoded by a single canine gene was established for a Transcript cluster for which gene annotation was unavailable (Table 2).

**Table 2:** Genes displaying ≥ 1.5-fold differential expression between 18 metastasising and 10 non-metastasising OMMs as measured by microarray analysis.

Gene description (Gene symbol/ID.)	Chromosomal location^b^	Gene-level fold change (NM/M)^C^	Adj_p-value^d^
PQ loop repeat containing 1 (*PQLC1*)	1: 0.76	1.72	0.023
Small nucleolar RNA SNORA61	2: 71.92	2.16	0.016
Small nucleolar RNA SNORD104	9: 12.02	1.50	0.030
Dolichyl-phosphate mannosyltransferase polypeptide 2, regulatory subunit (*DPM2*)	9: 55.49	1.51	0.050
Solute carrier family 25, member 51 (*SLC25A51*)	11: 54.16	1.61	0.016
Ribosomal protein L29 (*RPL29*)	20: 37.73	1.50	0.019
Small nucleolar RNA SNORA76	27: 36.79	1.70	0.017
Chemokine (C-X-C motif) ligand 12 (*CXCL12*)	28: 2.90	2.27	0.049
Disintegrin and metalloproteinase domain-containing protein 10 (ADAM10)	30: 23.61	0.66	0.029
RNA-Binding Motif Protein 3 (*RBM3*)	X: 41.81	1.62	0.013
Small nucleolar RNA SNORD61	X: 107.18	3.33	0.008
Sequence similarity to Apolipoprotein B mRNA editing enzyme catalytic subunit 3 A (E-val: 0.0; 375 bp; 88%) (*APOBEC3A*)^a^	Unknown	0.04	0.025

^a^Transcript cluster with no gene annotation. The most significant similarity between the sequence (spliced exons) of the Transcript cluster and a canine mRNA is listed. The significance of the sequence similarity is denoted by the E value and the length of the sequence alignment, and the proportion of the Transcript cluster sequence included in the alignment is stated.

^b^Chromosomal location is denoted by the chromosome name and the gene start base co-ordinate^55^.

^c^Ratio of median gene-level expression values.^d^Permutation testing-adjusted t-test p-value.

### Functional annotation enrichment analysis

For Transcript clusters for which an Ensembl Gene ID^55^ could be defined, the frequencies of functional annotations assigned to the Transcript clusters differentially expressed (300 of 331) between the M and NM OMMs were compared to those assigned to the Transcript clusters (11,842 of 13,422) for which the expression (above background) of ≥1 exon probe set was detected in at least 30% of the OMMs in the M and/or M groups. Over-represented amongst the genes exhibiting differential expression were 4 Gene Ontology Consortium biological processes and one KEGG pathway (Table 3).

**Table 3:** Differentially expressed gene-associated enriched functional annotations.

Functional annotation^a^	Fold enrichment^b^	P-value^c^	Gene expression	
			NM > M	M > NM
GO: 0010923 negative regulation of phosphatase activity	5.972	0.009	GPATCH2, PPP1R37	CASC5, CHP1, CSRNP2
cfa03460: Fanconi anaemia pathway	4.821	0.018	FANCC	FANCB, FANCI, RPA2, TOP3A
GO: 0000266 mitochondrial fission	11.824	0.025	COX10, MUL1	MTFR1
GO: 0010875 positive regulation of cholesterol efflux	10.749	0.030	NR1H3, PLTP	APOE
GO: 0042632 cholesterol homeostasis	5.086	0.042	NR1H3	ABCA2, APOE, MTTP

^a^GO BP: Gene Ontology Biological Process; KP: Kegg Pathway.

^b^Fold enrichment - Proportion of 300 differentially expressed genes with the functional annotation/proportion of 11,842 genes expressed in the OMM that have the functional annotation.

^c^P-value: Fisher Exact test p-value (EASE score) modified to reduce false positive results.

### RT-qPCR validation of differential expression

The expression, in the 18 M and 10 NM OMMs included in differential gene expression analysis, of the 12 genes which displayed >1.5-fold differences in expression between M and NM OMMs were assayed by RT-qPCR (Table 4). The expression of Small nucleolar RNA SNORD61 (which had the lowest median level of expression of the 12 differentially expressed genes) could not be measured reliably in the OMM samples (Cq values >35 were obtained, or amplification was not detected), although its expression could be detected (Geomean Cq = 27.14) in cDNA prepared from 2 µg of a pool of OMM total RNA samples.

**Table 4:** Differences in gene expression between M and NM OMMs measured by RT-qPCR.

Gene symbol/ID.	Exon-level fold change^a^ (NM/M)	RT-qPCR				
		No. NM OMMs^b^	No. M OMMs^b^	Fold change^c^ (NM/M)	Spearman RCC^d^	p-value^e^
*PQLC1*	9.26	10	17	1.32	0.52 (0.006)	0.94
SNORA61	2.16	10	17	1.76	0.63 (0.0004)	0.76
SNORD104	1.50	10	16	1.82	0.59 (0.001)	0.76
*DPM2*	1.90	9	18	1.34	0.49 (0.010)	0.94
*SLC25A51*	1.61	10	14	0.52	−0.35 (0.097)	0.31
*RPL29*	1.41	10	17	2.39	0.51 (0.006)	0.34
SNORA76	1.70	6	12	1.12	−0.1 (0.702)	0.94
*CXCL12*	31.08	10	17	7.14	0.43 (0.024)	0.04
*ADAM10*	0.42	10	17	0.96	0.41 (0.035)	0.76
*RBM3*	1.35	10	18	0.95	0.16 (0.425)	0.94
*APOBEC3A*	0.04	7	17	0.20	0.72 (0.00008)	0.08

^a^Fold change difference in expression between 18 M and 10 NM OMMs determined by microarray - Ratio of median expression values for the Exon probe set upon which RT-qPCR assay design was based.

^b^The numbers of NM and M OMMs represent the numbers of samples for which valid Cq (Cq < 35; Cq SD < 0.5) measurements were. obtained. ‘Non-valid’ Cq values were attributable to: Cq < 35 or ‘undetermined’ and Cq SD > 0.5.

^c^Fold change (ratio of median expression values) determined by RT-qPCR assay.

^d^The Spearman rank correlation coefficient (RCC) indicates the extent of the concordance between the expression values for individual OMMs assayed by microarray and RT-qPCR, respectively.The statistical significance (two-tailed p-value) of the correlation is shown in parenthesis.

^e^The statistical significance of differences between the RT-qPCR measured gene expression values for the NM and M OMMs determined by t-test.

For most of the other 11 genes, Cq values ≥35/’undetermined’ and/or a Cq standard deviation (triplicate assays) of >35 meant that valid gene expression measurements were recorded for slightly fewer than the 28 OMM biopsies profiled by microarray hybridisation. For 8 genes, the expression levels in individual OMMs measured by microarray (exon-level probe set) and RT-qPCR, respectively, were highly concordant, as were the NM OMM/M OMM fold differences in expression determined by the two techniques (Table 4). For SLC25A51 and SNORA76 there was a negative correlation between the gene expression values measured by microarray and RT-qPCR, and for SLC25A51 there was a difference between the ‘direction’ of NM: M differential expression as assessed by microarray and RT-qPCR, respectively (Table 4). These results suggest that for both SLC25A51 and SNORA76 the transcript quantified by PCR assay was different to that measured by microarray hybridisation. The differential expression of *CXCL12* achieved statistical significance.

### Class prediction analysis

**Figure 2:**
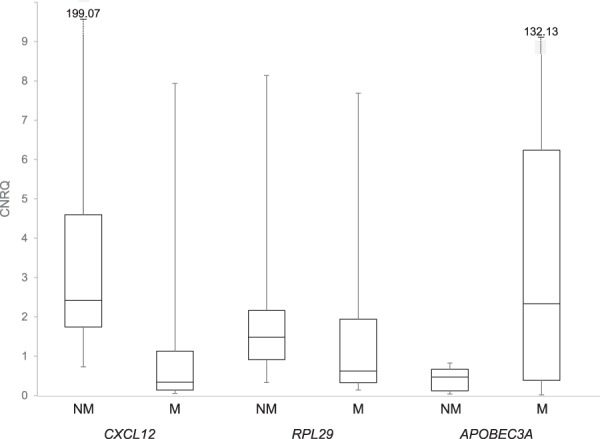
RT-qPCR-measured expression levels in OMMs of 3 genes employed in Linear Discriminant Analysis classifier. Expression values that encompass those shared by 25% and 75% of the OMMs are denoted by the bottom and top of each box, respectively. The median expression value is represented by the line within each box, and the maximum and minimum expression values are indicated by the lines extending above and below each box, respectively. M = metastasising tumour; NM = Non-metastasising OMM.

**Figure 3:**
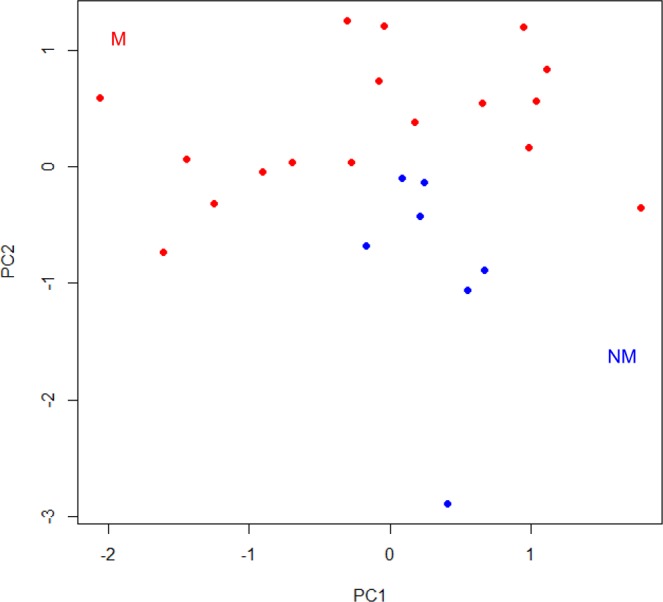
Relationship between M and NM OMMs in the context of the expression levels of the genes employed for class prediction. Principal component analysis was performed using the RT-qPCR-measured expression levels of *CXCL12*, *APOBEC3A* and *RPL29* in 17 M (red circles) and 7 NM (blue circles) OMMs. The first (PC1) and second (PC2) principal components are shown.

Based on an evaluation of the relative characteristics of the expression values measured for the 13,422 Transcript clusters ‘present’ in the OMMs, the optimal classification function for prediction of OMM ‘metastatic status’ (M or NM) was predicted to be Linear Discriminant Analysis (LDA; predicted accuracy = 0.761; lowest predicted accuracy - k-nearest neighbours = 0.408). The ranking of genes for their utility in class prediction may be based on the statistical significance of their difference in expression between classes. However, ‘filter’ gene selection methods may be based upon other metrics^56^, including fold-change differences in gene expression between classes^57,58^. Consequently, the efficacy of using the 3 genes shown (by RT-qPCR analysis) to exhibit >two-fold differential expression (Table 4, Fig. 2) for class prediction was tested. The relationships between the M and NM OMMs, in the context of the variation in the expression levels of *CXCL12*, *APOBEC3A* and *RPL29*, is effectively visualised by principal component analysis (Fig. 3).

**Figure 4:**
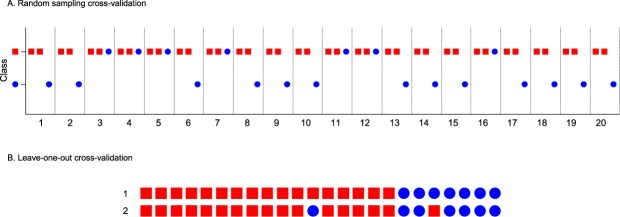
Class Prediction by Linear Discriminant Analysis. (**A**) Random sampling cross-validation. On each of 20 occasions, the RT-qPCR-measured expression values of 3 genes (*CXCL12*, *RPL29* and *APOBEC3A*) which displayed >two-fold differential expression between M and NM OMMs were used to predict the class (M = a square, and NM = a circle) of 3 randomly-selected OMMs (2 M OMMs and 1 NM OMM, which represent 10% of the OMMs and constituted a ‘test set’). Prior to class prediction, the LDA classifier was trained using the gene expression data obtained for the remaining 90% of the OMMs (15 M and 6 NM OMMs, which constituted a ‘training set’). (**B**) Leave-one-out cross-validation. The RT-qPCR-measured expression values of 3 genes (*CXCL12*, *RPL29* and *APOBEC3A*) which displayed >two-fold differential expression between M and NM OMMs were used to predict the class of each of 24 OMMs (17 M = squares, 7 NM = circles). On each of 24 occasions, the class of one OMM was predicted after the LDA classifier had been trained using the gene expression data obtained for the remaining 23 OMMs. Row 1 depicts the actual class of each OMM and row 2 the predicted class of each tumour.

Random sampling cross-validation was initially employed to test the performance of the LDA classifier. Two M OMMs and 1 NM OMM were randomly selected on each of 20 occasions, and the accuracy of their classification (as N or NM) measured after training the classifier using the remaining 15 M and 6 NM OMMs’ expression values (Fig. 4). For M OMMs, mean and median classification accuracies of 100% were estimated, whilst mean and median classification accuracies of 65% and 100%, respectively, were estimated for the NM OMMs. In a subsequent evaluation of classifier performance by ‘leave-one-out cross validation’, 94% of 17 M OMMs and 86% of 7 NM OMMs were correctly classified (Fig. 4).

## Discussion

Malignant melanomas are the most common canine melanocytic neoplasm^3,4,18^ and the most common oral malignancy in dogs^1^. At the present time there is no means to accurately predict if an individual OMM is one of the significant proportion of tumours that will metastasise, and metastasis is commonly underestimated during tumour staging due to a failure to sample all appropriate lymph nodes, and/or a failure of detection by standard cytology or histopathology. Metastasis is the most common cause of death in dogs treated for OMM, but there is currently no proven effective therapy to delay or prevent metastasis. With no means of accurately predicting OMM metastasis, it is possible that palliative adjuvant treatment is not prescribed for dogs with an unrecognised ‘metastasising OMM’.

Molecular genetic and epigenetic ‘events’ that promote canine OMM metastasis may be both predictive indicators of canine OMM metastasis and the focus for therapeutics intended to prevent metastasis. Through comparative genome-wide gene expression profiling of 18 primary OMMs that metastasised and 10 primary OMMs that did not metastasise, in the present study we aimed to identify dog breed-independent OMM metastasis-associated gene expression.

Increased expression in M OMMs characterised 60% of the genes differentially expressed between M and NM OMMs. Further indication of the potential significance to OMM metastasis of the ‘up-regulation’ of pro-metastatic gene expression is that a similar proportion of the genes differentially expressed between human oral mucosal melanoma lymph node metastases and paired non-metastatic lymph nodes were expressed at an increased level in the lymph node metastases^47^. An understanding of the mechanisms by which changes in gene expression mediate OMM metastasis is afforded by functional annotation enrichment analysis.

Reversible protein phosphorylation is integral to intracellular signal transduction pathways, and requires the coordinated action of protein kinases and protein phosphatases. Disruption of the balance between phosphorylation and dephosphorylation is associated with carcinogenesis, and protein phosphatases have been recognised as tumour suppressors^59^. Aberrant expression of protein phosphatase inhibitors has been reported in a wide variety of human cancers^60^. Two genes (*GPATCH2*, *PPP1R37*) with the ‘negative regulation of phosphatase activity’ annotation showed decreased expression in the M OMMs and three genes (*CASC5*, *CHP1*, *CSRNP2*) with the same annotation showed increased expression in the M OMMs. *PPP1R37*, *GPATCH2* (also known as *PPP1R30*), CASC5 (or *PPP1R55*) and *CSRNP2* (or *PPP1R72*) encode proteins recognised as inhibitory regulatory subunits of phosphoprotein phosphatase 1 (PPP1)^61^, a protein serine-threonine phosphatase that regulates several members of the Transforming growth factor beta signalling pathway^62^, which promotes invasion and metastasis in advanced stages of cancer^63^. Inhibition of PPP1 by the regulatory subunit PPP1R1A in Ewing sarcoma has been shown to promote tumour growth and metastasis^64^. CHP1 (Calcineurin Homologous Protein 1) inhibits the serine/threonine protein phosphatase 2B^65^, which is responsible for the dephosphorylation (and thus activation) of Nuclear factor of activated T cells (NFAT) transcription factors 1–4^66^. NFATs have been shown to have both pro-metastatic^67^ and anti-metastastic^68^ activities.

The Fanconi anaemia (FA) pathway is a multi-protein DNA repair pathway that resolves DNA interstrand cross-links encountered during DNA replication that would otherwise block replication and transcription, and lead to gross chromosome abnormalities^69^. The pathway forms part of the DNA damage response network^70^ which maintains genome integrity. Four genes (*FANCB*, *FANCI*, *RPA2*, *TOP3A*) with the ‘FA pathway’ annotation showed elevated expression in M OMMs, whilst a fifth FA pathway gene (*FANCC*) displayed marginally decreased expression in the M OMMs. FANCB and FANCC encode proteins that form part of the FA core complex, an ubiquitin E3 ligase that monoubiquinates FANCI (and FANCD2), the ID complex, which is subsequently re-localised to the DNA lesion, and (with Group III FA proteins) co-ordinates DNA cross-link repair^71^. The RPA2 and TOP3A proteins perform DNA repair functions associated with the FA pathway. RPA2 is a subunit of the Replication Protein A complex, which is involved in DNA repair in the cellular response to DNA damage^72^, whilst TOP3A encodes a DNA topoisomerase that controls DNA topology during DNA repair^73^. The elevated expression of DNA repair pathway genes in human primary cutaneous melanoma has been associated with distant metastasis and poor prognosis^74^. Subsequently, the increased expression of FA and DNA damage response pathway genes has been reported in human ‘high grade’ primary cutaneous melanoma (which are associated with significantly reduced survival), relative to ‘low grade’ primary tumours^45^, and in human cutaneous melanoma metastases relative to normal skin^75^. As FA pathway gene expression correlates with FA pathway activity^76^, it appears that the FA pathway is activated in metastasising human cutaneous melanoma and canine OMM. FA pathway activation may be a response to increased genome instability in advanced melanomas, and may confer a selective advantage supporting metastasis to distant sites.

Two genes (*COX10*, *MUL1)*) with the ‘mitochondrial fission’ annotation displayed decreased expression in the M OMMs and one gene (*MTFR1*) with the same annotation showed elevated expression in the M OMMs. Increased expression of mitochondrial fission pathway genes has been associated with the invasiveness and metastasis of some cancers^77,78^. *MTFR1* (Mitochondrial Fission Regulator 1) is upregulated in metastatic uveal melanoma^79^, and is a member of a 20-gene panel whose collective high expression is predictive of prostate cancer metastasis^80^. Conversely, suppression of *MUL1* (Mitochondrial E3 Ubiquitin Protein Ligase 1) has been associated with the progression of human head and neck cancer^81^. *COX10* (Cytochrome C Oxidase Assembly Homolog 10) is a member of a 14-gene classifier for colorectal cancer metastasis, identified by differential gene expression analysis of early and late stage primary colorectal cancer^82^.

Deregulation of the expression of genes involved in cholesterol homeostasis pathways has been associated with cancer development and progression^83^. In melanoma, the increased expression of 7 cholesterol synthesis pathway genes has been correlated with decreased patient survival^84^. Two genes (*NR1H3*, *PLTP*) with the ‘Positive regulation of cholesterol efflux’ and/or ‘(positive regulation of) cholesterol homeostasis’ annotation were expressed at a decreased level in the M OMMs and three genes (*APOE*, *MTTP*, *ABCA2*) with one, or both, of the same annotations, showed elevated expression in the M OMMs. *NR1H3* (or Liver X Receptor Alpha isoform) is a Nuclear Receptor superfamily transcription factor which when activated by oxysterol binding drives cholesterol efflux^85^. Agonist activation of the Liver X Receptor Beta isoform (*NR1H2*) has been shown to suppress the growth and metastasis of melanoma cells by transcriptional induction of apolipoprotein-E^86^. Reduced *NR1H3* expression is predictive of decreased recurrence-free survival in muscle-invasive bladder cancer^87^, and associated with reduced overall survival in hepatocellular carcinoma^88^. *APOE* (apolipoprotein-E) is a lipid transport protein essential for the normal catabolism of triglyceride-rich lipoproteins^89^. Elevated APOE expression is associated with lymph node metastasis of human gastric cancer^90^ and lung adenocarcinoma^91^, although it has been identified as a metastasis suppressor *in vitro* in human cutaneous melanoma^92^. *PLTP* (Phospholipid Transfer Protein) transfers phospholipids from triglyceride-rich lipoproteins to high density lipoprotein, and is involved in the uptake of cholesterol from peripheral cells and tissues. *PLTP* expression was increased in Grade IV human glioma relative to low grade glioma, and knockdown *in vitro* lead to the decreased migration of glioblastoma tumour cells^93^. In concept, the increased expression of *PLTP* in the NM OMMs observed in the present study may be consistent with the production by the tumours of interleukin 6, which has been shown to inhibit melanoma growth^94^. The increased expression of *PLTP* in the spontaneous regression phase of canine transmissible venereal tumour has previously been associated with increased IL-6 production^95^. The ATP-binding cassette transporter 2 (ABCA2) is a membrane-associated protein involved in sphingolipid transport. ABCA2 deficiency inhibits prostate tumour metastasis *in vivo*, potentially through reduction of the intracellular sphingolipid level^96^, whilst ABCA2 expression is increased in ovarian carcinoma metastases relative to primary tumours^97^.

In the present study, RT-qPCR analysis confirmed >two-fold differential expression between M and NM OMMs for 3 genes (*CXCL12*, *APOBEC3A* and *RPL29*). As fold change has been effectively employed to rank genes for their potential efficacy in gene expression level-based classification^57,58^, the 3 genes were selected for use in class prediction. Cross-validation was deployed in a preliminary evaluation to test the accuracy of a Linear Discriminant Analysis-based classifier featuring the 3 genes. The classification accuracies estimated were 94–100% (M OMMs) and 86–100% (NM OMMs), respectively. The LDA classifier performance will need to be validated by further retrospective, and prospective study. If the classification accuracy is confirmed, the cross-breed OMM metastasis-associated 3-gene expression signature would form the basis of an objective and quantitative predictive test for OMM metastasis that could make a significant contribution to the clinical management of canine OMM.

*CXCL12* (C-X-C Motif Chemokine Ligand 12, or stromal cell-derived factor-1) is secreted by stromal cells and is a ligand for the G-protein coupled receptors CXCR4 and CXCR7^98^. Binding of CXCL12 to CXCR4 activates four signal transduction pathways that induce cytoskeletal rearrangement, cell growth, angiogenesis, and anti-apoptotic effects^98^. Interaction between CXCL12 and CXCR4 has also been shown to mediate metastasis, and direct metastatic dissemination to organs expressing high levels of *CXCL12*^99^. Blocking CXCL12 binding to CXCR4 reduced the migration of human uveal melanoma cells *in vitro*^100^, and pulmonary metastasis of murine B16 cutaneous melanoma cells^101^. Low *CXCL12* expression in human primary cutaneous melanomas has been associated with poor prognosis^102^ and shown to be predictive of metastasis^103^, and *CXCL12* is one of 789 genes displaying reduced expression in ‘high grade’ human primary cutaneous melanomas that is a member of a 1,864 gene expression signature that delineates two classes of primary cutaneous melanomas with significantly different rates of metastasis^104^.

*APOBEC3A* (Apolipoprotein B mRNA Editing Enzyme Catalytic Subunit 3A) encodes a cytidine deaminase which preferentially binds to the sequence 5′-(C/T)TCA in RNA or single-stranded DNA and converts cytosine to uracil. Deregulated *APOBEC3A* expression in cancer is believed to induce double strand breaks in genomic DNA activating DNA damage response pathways^105^. The repair of such breaks triggers the formation of single stranded DNAs which are substrates for *APOBEC3A*-mediated hypermutation, such that 5′-(C/T)TTA *APOBEC3A* mutation signatures occur in clusters (on one DNA strand) in multiple human cancers^106^. The extent of APOBEC-associated mutations correlates with APOBEC mRNA expression levels^107^. *APOBEC3A*-mediated mutagenesis occurs at different stages in different cancers^108^, and is thought to drive tumour evolution, including promoting metastasis^109^.

*RPL29* (Ribosomal protein L29) encodes a component of the 60 S ribosomal subunit. Beyond their role in ribosome assembly and protein translation, differential expression of ribosomal protein genes in cancer has been associated with ribosome-independent regulation of cell growth and proliferation, apoptosis, invasion and metastasis^110^. Ribosome-free ribosomal proteins have been implicated as being both oncogenic and tumour-suppressors^110^. Silencing of *RPL29* suppressed the proliferation of human pancreatic tumour cells and enhanced apoptosis^111^ suggesting an involvement in cell proliferation. However, *RPL29* silencing had no effect on the viability of human metastatic melanoma cells^112^. The expression of a specific ribosomal protein has been shown to be a prognostic indicator for multiple human cancers^113^.

In human medicine, anti-angiogenics and matrix metalloproteinase inhibitors have been licenced for treatment of tumour metastasis^114^. Cellular receptors in signal transduction pathways that control cell to cell and cell to ECM adhesion are targets for anti-metastatics in development^115^. Pro-metastatic gene expression in OMMs is a potential target for anti-metastasis therapeutics. Targeting the interaction between the CXCR4 receptor and CXCL12 has been evaluated as a strategy for inhibiting CXCR4-CXCL12 axis-mediated melanoma metastasis. Small molecule inhibitors of CXCR4 were shown to be effective at disrupting the liver metastasis of uveal melanoma cells in mice^116^, and migration of human cutaneous melanoma cells *in vitro*^117^. Chemical inhibition of the mutational activity of APOBEC3A (and APOBEC3B) is being evaluated as a cancer therapeutic^118^. Furthermore, demonstration of microRNA post-transcriptional regulation of APOBEC gene expression^119^ suggests the use of miR-mimics^120^ as a potential means of APOBEC deaminase inactivation^119^. Intriguingly, it has been postulated that APOBEC3A-mediated hypermutation could generate new tumour-specific antigens thereby enhancing the efficacy of immune stimulation therapies^106^. The potential for suppressing melanoma metastasis through activation of Liver X Receptors (LXR) using synthetic agonists has been investigated. Activation of the LXR Beta isoform (*NR1H2*) was shown to inhibit human and murine cutaneous melanoma cell migration *in vitro* and murine cutaneous melanoma cell metastasis in a mouse xenograft model^86,121^. If *NR1H3* is the predominant LXR Receptor isoform expressed it remains to be seen if LXR agonists would initiate receptor activation with anti-metastatic effect. The up-regulation of FA pathway DNA repair genes in human cutaneous melanomas and canine OMM may be both pro-metastatic by negating the impact of increased genome instability, and contribute to melanoma metastases chemoresistance. Consequently, in concept, the use of FA pathway inhibitors^122^ may be an option for treatment of melanoma.

The major limitation of this study was that the number of NM OMMs included was restricted by the need to attempt to ensure that a primary tumour classified as ‘non-metastasising’ was only classified as such because it did not exhibit pro-metastatic gene expression as opposed to its metastasis potentially being prevented by a systemic adjuvant therapy. The consequence of the widespread use of the xenogenic melanoma vaccine in the UK was the exclusion of OMM biopsies from many dogs that had not developed metastatic disease. Furthermore, due to difficulties associated with collecting fresh canine tumour biopsies, FFPE biopsies of primary canine OMM (surgically removed at specialist veterinary oncology centres) were used in the study. However, data that is both biologically authentic and clinically-relevant has been obtained by Affymetrix microarray-based gene expression profiling of FFPE tissues^123,124^. Validation of the predictive accuracy of the 3-gene LDA classifier will be achieved through further retrospective, and prospective, studies featuring larger numbers of (optimally freshly collected) M and NM OMM biopsies. Ultimately, experimental investigations involving canine OMM cells will be necessary to confirm the functional consequences (e.g. in regard to cell migration) of metastasis-associated differential gene expression that are anticipated given gene function(s) and (in some cases) prior *in vitro* and *in vivo* study evidence.

Several of the differences in gene expression observed between primary canine OMMs that metastasised and OMMs that did not metastasise in this study have previously been associated with human cutaneous melanoma metastasis. The genes involved have been targets in proof of principle trials of potential anti-metastatic melanoma therapeutics. Other genes that exhibit differential expression between metastasising and non-metastasising primary canine OMMs may represent potential new targets for both canine and human cancer drugs. The results obtained in the present study suggest that OMMs in dogs may be responsive to anti-human melanoma metastasis therapeutics currently in clinical trials, or being evaluated through preclinical *in vitro* and *in vivo* model studies, and may be as likely as human melanomas to be responsive to therapeutics whose efficacy for treatment of human melanoma has yet to be investigated. Quantification of the expression of 3 genes, each of which displays a greater than two-fold differential expression between canine OMMs that do and do not metastasise, may be the potential basis for a test that would accurately predict canine OMM metastasis, and thereby assist a clinician to make an informed decision about the most appropriate treatment for a canine OMM patient.

## Materials and Methods

### Ethics Statements

This study was conducted with the approval of the ethics committees of the Animal Health Trust and the University of Liverpool, respectively. All methods were performed in accordance with the relevant guidelines and regulations. An OMM biopsy was only included in the study with the informed, written consent of the owner of the dog who bore the tumour. The treatment that a OMM patient received was unaffected by the inclusion of a biopsy of their tumour in the study.

### Tumour Samples

FFPE biopsies of OMMs were collected (between 1993 and 2010) for histopathology from dogs attending the Clinical Oncology departments at the Animal Health Trust Centre for Small Animal Studies, University of Liverpool Small Animal Teaching Hospital, Dick White Referrals, and Colorado State University Veterinary Teaching Hospital. Biopsies were recovered by surgery performed (prior to any adjuvant treatment) on dogs for whom complete staging and follow-up information were available. Metastasis was confirmed by abdominal ultrasound or computed tomography, and cytological/histological examination of ≥1 regional lymph nodes. ‘Metastasising’ (M) OMM biopsies were from dogs that had pathological analysis *and* diagnostic imaging-confirmed metastasis, *and* who died or were euthanased because of OMM metastasis <500 days after surgery/biopsy (irrespective of adjuvant chemotherapy, including prednisolone, xenogeneic vaccine, and/or radiotherapy). ‘Non-metastasising’ (NM) OMM biopsies were from dogs without pathological analysis and imaging-confirmed metastasis, whom did not receive any adjuvant therapy, *and* who were still alive >540 days post-surgery/biopsy.

### RNA isolation and purification

The RecoverAll Total Nucleic Acid Isolation Kit, which facilitates on-column DNase digestion (ThermoFisher Scientific, Paisley, UK), was used to isolate total RNA from FFPE OMM biopsies. RNA samples were further purified by spin column filtration (OneStep PCR Inhibitor Removal Kit; Zymo Research, Freiburg, Germany) and additional DNase treatment (TURBO DNA-free kit; ThermoFisher Scientific, Paisley, UK). Finally, RNAs were concentrated (RNA Clean & Concentrator-5; Zymo Research, Freiburg, Germany) and quantified by RiboGreen fluorometry (Quant-iT RiboGreen RNA Assay Kit, ThermoFisher Scientific, Paisley, UK).

### RNA sample selection

Reverse transcription-quantitative PCR (RT-qPCR) measurement of a 126 bp fragment of a 130–150 bp short interspersed nuclear element (SINE) that occurs every 5–8.3 kb in the canine genome^125^ was employed to assess the integrity of each FFPE RNA sample as described previously^50^. Procedural details are summarised in the Supplementary Information.

### Genome-wide gene expression profiling

#### RNA amplification, labelling and microarray hybridisation

Fragmented, biotinylated single-stranded cDNA was prepared from 5.1 ng of each FFPE OMM RNA sample using the GeneChip WT Pico Reagent Kit (ThermoFisher Scientific, Paisley, UK). cDNA preparation involved target amplification by 11 cycles of adaptor-primer PCR and 14 h of *in vitro* transcription. Each cDNA was individually hybridised to an array in a Canine Gene 1.1 ST Array Strip (ThermoFisher Scientific, Paisley, UK), in a proprietary hybridisation cocktail (ThermoFisher Scientific, Paisley, UK), for 20 h at 48 °C. Array strip washing and streptavidin-phycoerythrin staining were undertaken by the GeneAtlas System (ThermoFisher Scientific, Paisley, UK) Fluidics Station, and array scanning by the GeneAtlas System (ThermoFisher Scientific, Paisley, UK) Imaging Station.

#### Microarray data analysis

Microarray data was processed using the ‘Affymetrix Expression Console Software 1.3’ (ThermoFisher Scientific, Paisley, UK). ‘Outlier arrays’ were first identified by review of exon-level probe set expression values created (using the RMA algorithm^126^) by quantile normalisation, log_2_ transformation and signal summarisation, respectively. Arrays with ≥1 sample quality, labelling quality or hybridisation quality metric value ≥2 standard deviations from the mean for all the arrays^54^ were excluded, and the raw probe-level signal intensity data for the remaining arrays re-processed to generate quantile normalised and log_2_-transformed exon and gene-level probe set expression values. Gene-level probe sets (‘Transcript clusters’) annotated as ‘crosshyb_type’ = 1 (unique hybridisation target) and ‘category’ = ‘main’^127^, and for which the expression above background (detection above background p-value < 0.01^128^) of ≥l exon probe set could be detected in at least 30% of the OMMs in the M and/or NM group, were judged to be expressed in the OMMs and were included in further analyses.

Hierarchical clustering (average linkage; similarity metric = Pearson Correlation Coefficient) was performed using Cluster^129^, and Principal Component Analysis using the R stats package function *prcomp*^130^, in order to view the relationships between OMMs on the basis of their gene-level expression profiles. A two-tailed t-test for unpaired data was employed to identify genes exhibiting differential expression between M and NM OMMs that was statistically significant, adjusting P-values by permutation testing^131^ to correct for false positives arising from multiple testing. BLAST similarity search (against canine and human mRNAs and non-coding RNAs) was employed to attempt to establish the potential identity of Transcript clusters representing ‘predicted genes’, or for which gene annotation was unavailable.

#### Functional annotation analysis

The biological processes and pathways affected by the differences in gene expression observed between M and NM OMMs were identified using DAVID^132,133^. The functional annotations associated with differentially expressed genes were compared with those ascribed to all Transcript clusters (‘crosshyb_type’ = 1 and ‘category’ = ‘main’) for which the expression of ≥1 exon probe set was detected above background in ≥30% of the tumours in the NM and/or M OMM cohort, and over-represented biological processes and pathways identified.

### Reverse transcription-quantitative PCR (RT-qPCR)

RT-qPCR was employed for validation of differential gene expression. A unique region within the exon probe set that displayed the largest statistically significant fold-difference in expression between M and NM OMMs was the template for design (using Beacon Designer; Premier Biosoft, Palo Alto, USA) of a TaqMan or SYBR Green PCR assay. The expression level of each gene in each OMM sample was measured as the geometric Cq value calculated from triplicate PCR reactions performed using preamplified cDNAs, prepared from cDNAs previously screened for PCR inhibitors. A 71 bp fragment of a SINE^125^, present in the 3′-untranslated region of hundreds of canine mRNAs, was also assayed as a ‘reference gene’ for target gene expression measure normalisation^134^. Geometric mean Cq ≥35 were considered an unreliable measurement of gene expression and were excluded, as were the results of triplicate PCR assays with a Cq standard deviation of >0.5. Additional information is provided in the Supplementary Information.

#### RT-qPCR data analysis

Relative quantification of gene expression was performed using a modification of the delta-delta-Ct method which accounts for differences between the amplification efficiencies of target and potentially multiple ‘reference’ genes^135^. Using qbase + (Biogazelle, Gent, Belgium), target gene geometric mean Cq values were converted to relative gene expression measurements (‘Normalised Relative Quantity; NRQ^135^) by application of a canine SINE^125,134^ geometric mean C_q_-derived normalisation factor. A two-tailed t-test for unpaired data was performed on NRQ log_10_ transformations to identify genes exhibiting statistically significant differences in expression between M and NM OMMs.

### Class prediction analysis

The R package SPreFuGED^136^ predicts the performance in class prediction of representatives of 10 classification functions and, through evaluation of the expression values obtained for the Transcript clusters expressed in the OMM, was employed to identify the optimal classification function for prediction of OMM ‘metastatic status’ (M or NM) on the basis of OMM gene expression. Linear Discriminant Analysis-based class prediction was undertaken using the R Package MASS^130^ *lda* function implemented in the R environment for statistical analyses^137^. The accuracy of class prediction, performed using RT-qPCR-derived gene expression measurements, was estimated by cross-validation. In random sampling cross-validation, on each of 20 occasions the class (M or NM) of two M and one NM OMM, which constituted a ‘test data set’, were predicted after the classifier had been trained using the gene expression values (‘training data set’) obtained for the remaining OMMs (90% of the tumours). In leave-one-out cross-validation, the class of each OMM was predicted after the classifier had been trained using the remaining (n-1) OMM gene expression data.

## Supplementary information

Supplementary Information

## Data Availability

The microarray gene expression data generated during this study is available from the Gene Expression Omnibus repository (https://www.ncbi.nlm.nih.gov/geo/query/acc.cgi?acc=GSE129750).
