# Supplementary material for: Genome-wide analysis of canine oral malignant melanoma metastasis-associated gene expression
Source: Sci Rep. 2019 Apr 24;9:6511. doi: 10.1038/s41598-019-42839-x (PMC6482147; doi:10.1038/s41598-019-42839-x)
Supplement: Supplementary file 1 — Supplementary Information [file 41598_2019_42839_MOESM1_ESM.docx]

**Supplementary Information**

Title: **Genome-wide analysis of canine oral malignant melanoma metastasis-**

**associated gene expression**

Bowlt Blacklock, KL^1^ , Birand, Z^1^, Selmic, LE^2^, Nelissen, P^3^, Murphy, S^1#a^, Blackwood, L^4^, Bass, J^1#b^, McKay, J^5^, Fox, R^6^, Beaver, S^7^, Starkey, M^1^*

^1^Animal Health Trust, Newmarket, Suffolk, UK

^2^Department of Veterinary Clinical Sciences, The Ohio State University, Columbus, Ohio, USA

^3^Dick White Referrals, Newmarket, Suffolk, UK

^4^Institute of Veterinary Science, University of Liverpool, UK

^5^IDEXX Laboratories, Ltd, Wetherby, UK

###### ^6^Finn Pathologists, Harleston, UK

^7^Nationwide Laboratory Services, Poulton-le-Fylde, UK

^#a^Current address: The Royal (Dick) School of Veterinary Studies, University of Edinburgh, Edinburgh, UK

###### ^#b^Current address: Finn Pathologists, Harleston, UK

*Corresponding author

E-mail: mike.starkey@aht.org.uk

**Materials and Methods**

RNA sample selection

The integrity of each formalin-fixed, paraffin-embedded (FFPE) oral malignant melanoma (OMM) RNA sample was assessed by reverse transcription-quantitative PCR (RT-qPCR) assay of a 126bp fragment of a 130 - 150bp short interspersed nuclear element (SINE) present every 5 - 8.3kb in the canine genome^1^, and shown (by BLAST similarity search) to be present in the 3’-untranlsated region of hundreds of canine mRNAs. cDNA was prepared from 10ng of each total RNA sample using the High-Capacity cDNA Reverse Transcription Kit (ThermoFisher Scientific, Paisley, UK). Each OMM cDNA (18µl) was diluted with 10µl of 1mM Tris-HCl (pH 8.0), 0.1mM EDTA and 1µl used in triplicate 10µl PCR reactions, comprising 1 x PowerUp SYBR Green Master Mix (ThermoFisher Scientific, Paisley, UK) and 0.3µM of each of the CfSINE126 PCR primers (Table S4A). The PCR reaction master mix was UV irradiated at 302nm for 5 min prior to addition of the CfSINE126 PCR primers. Thermocycling (ABI StepOne Plus; ThermoFisher Scientific, Paisley, UK) was performed as follows: 50°C, 2 min; 95°C, 2 min; (95°C, 3s; 60°C, 30s) x 40; melt curve program.

Reverse transcription-quantitative PCR (RT-qPCR)

*Reverse transcription*

cDNA was prepared (in a 20µl reaction) from 15ng of each OMM RNA using the High-Capacity cDNA Reverse Transcription Kit (ThermoFisher Scientific, Paisley, UK).

*Assay for reverse transcription and PCR inhibitors*

Prior to use in RT-qPCR assays, each OMM cDNA was screened for the presence of PCR (and potentially reverse transcription) inhibitors. An aliquot of each OMM cDNA was spiked with an equal amount of a synthetic *solanum tuberosum* (SPUD)-derived amplicon^2^, and the SPUD amplicon quantification cycle (Cq) value obtained for each upon PCR amplification compared with that measured for the SPUD amplicon in the absence of OMM cDNA. Duplicate 10µl PCR reactions were performed for each OMM cDNA, and comprised 1.5µl of cDNA, 1 x PowerUp SYBR Green Master Mix (ThermoFisher Scientific, Paisley, UK), 1.33fM of SPUD amplicon (Table S4B), and 0.3µM of forward and reverse SPUD primers (Table S3B). In duplicate ‘SPUD amplicon alone reactions’, an OMM cDNA was replaced with 1.5µl of nuclease-free water. PCR reactions were run in an ABI StepOne Plus machine (ThermoFisher Scientific) using the following program: 98°C, 2min; (98°C, 5s; 60°C, 30s) x 40; melt curve program.

*cDNA preamplification*

An 11µl aliquot of each OMM cDNA was preamplified in a 50µl reaction, comprising 1 x TaqMan PreAmp Master Mix (ThermoFisher Scientific, Paisley, UK) and 45nM of both the forward and reverse PCR primer for each gene to be subsequently assayed. Preamplification was performed on a DNA Engine Tetrad (Bio-Rad) at 95°C for 10 min, followed by 14 cycles of (95°C for 15s and 60°C for 4 min). A 45µl aliquot of each preamplified cDNA was diluted with 855µl of nuclease-free water, and 2.5µl aliquots were used for quantitative PCR.

*Quantitative PCR*

Triplicate PCR reactions were performed for each preamplified OMM cDNA. TaqMan PCR reactions (10µl) comprised 1 x TaqMan Gene Expression Master Mix (ThermoFisher Scientific, Paisley, UK) and the TaqMan assay reagents (Table S4C). Thermocycling (ABI StepOne Plus; ThermoFisher Scientific, Paisley, UK) was performed as follows: 50°C, 2 min; 95°C, 10 min; (95°C, 15s; 60°C, 1 min) x 40. SYBR Green PCR reactions (10µl), comprising 1 x PowerUp SYBR Green Master Mix (ThermoFisher Scientific, Paisley, UK) and forward and reverse PCR primers (Table S4C), were thermocycled (ABI StepOne Plus; ThermoFisher Scientific, Paisley, UK) as follows: 50°C, 2 min; 95°C, 2 min; (95°C, 3s; 60°C, 30s) x 40; melt curve program.

**Results**

Microarray data analysis

*Identification of ‘outlier arrays’*

Four arrays (GS1, SB1, LR6 and A1) that had ≥1 ‘RNA sample quality metric’^3^ 2 standard deviations away from the mean of the metric value for the sample cohort were flagged as outliers^4^ (Table S3) and excluded.

Reverse transcription-quantitative PCR (RT-qPCR)

*Assay for reverse transcription and PCR inhibitors*

All the OMM RNAs had a higher mean Cq than the SPUD amplicon alone (median difference = +0.24 cycles for M OMMs and +0.17 cycles for the NM OMMs; Table S5). However, the extent of the putative inhibitor-related increase in the SPUD amplicon Cq values measured in the presence of the OMM RNAs is similar for both the M and NM OMMs (the median M and NM OMM group Cq values differ by 0.07, and the mean M and NM OMM group Cq values differ by 0.04) (Table S5). Differences of this magnitude will be adequately adjusted for by normalisation when generating relative expression values. Furthermore, the differences between the SPUD amplicon Cq values measured in the presence and absence of a OMM RNA (<0.5 cycles, which is often used as the permissible Cq standard deviation for replicate qPCR assays) do not suggest that inhibition of reverse transcription and/or PCR would significantly affect RT-qPCR assay sensitivity.

**References**

1. Das, M. *et al*. Characterization of an abundant short interspersed nuclear element (SINE) present in *Canis familiaris*. *Mamm. Genome* **9**, 64-69 (1998).
2. Nolan, T., Hands, R.E., Ogunkolade, W. & Bustin, S.A. SPUD: a quantitative PCR assay for the detection of inhibitors in nucleic acid preparations. *Anal Biochem.* **351**, 308-310 (2006).
3. Affymetrix White Paper: Quality Assessment of Exon and Gene Arrays. Revision Date: 2007‐04‐06, Revision Version: 1.1. https://assets.thermofisher.com/TFS-Assets/LSG/ brochures/exon_gene_arrays_qa_whitepaper.pdf.
4. QC Metrics for Exon and Gene Design Expression Arrays. A summary based on the Affymetrix Quality Assessment of Exon and Gene Arrays White Paper. http://static1.1.sqspcdn.com/static/f/1438485/21486054/1359060361517/qc_metrics_

exon_gene_qrc.pdf.

**Tables**

Table S1. Dogs with an oral malignant melanoma included in the research study

| **Dog ID.** | **OMM status**^a^ | **Melanoma stage**^b^ | **Surgery** | **Radiation therapy** | **Additional treatment**^d^ |
| --- | --- | --- | --- | --- | --- |
| D1 | M | III | No | No | Meloxicam |
| LR1 | M | IV | Yes | Yes | Carboplatin |
| CB1 | M | III | Yes | Yes | Carboplatin |
| CB2 | M | IV | Yes | Yes | Carboplatin |
| LR2 | M | III | No | No | Meloxicam |
| D2 | M | IV | No | Yes | None |
| LR3 | M | IV | No | Yes | None |
| GR1 | M | III | No | Yes | Melanoma vaccine, COP |
| CS1 | M | IV | No | No | Melanoma vaccine |
| GR2 | M | III | No | No | Meloxicam |
| CB3 | M | III | No | No | None |
| CB4 | M | III | No | No | None |
| CB5 | M | IV | Yes | No | None |
| GR3 | M | III | No | No | None |
| GR4 | M | III | No | No | None |
| BM1 | M | III | No | No | None |
| GS1 | M | III | No | No | None |
| CB6 | M | III | No | No | Meloxicam |
| SB1 | M | III | Yes | No | None |
| BC1 | M | IV | No | No | None |
| GD1 | NM | III^c^ | Yes | No | None |
| LR6 | NM | III^c^ | Yes | No | None |
| GR5 | NM | I | Yes | No | None |
| GR6 | NM | I | Yes | No | None |
| LR4 | NM | II | Yes | No | None |
| A1 | NM | I | Yes | No | None |
| LR5 | NM | I | Yes | No | None |
| GR7 | NM | I | Yes | No | None |
| IT1 | NM | I | Yes | No | None |
| BF1 | NM | III^c^ | Yes | No | None |
| GR8 | NM | II | Yes | No | None |
| CB7 | NM | II | Yes | No | None |

^a^M - OMM metastasised; NM - OMM did not metastasise.

^b^Melanoma stage at initial presentation to the referral hospital. World Health Organisation staging scheme for canine oral melanoma: Stage I = <2cm diameter tumour, Stage II = 2cm to <4cm diameter tumour, Stage III = ≥4cm diameter tumour and/or lymph node metastasis; Stage IV = distant metastasis.

^c^Stage III melanoma due to ≥4cm diameter tumour.

^d^COP - Cyclophosphamide, vincristine and prednisone combination chemotherapy.

Table S2. Primary oral malignant melanoma RNAs used for gene expression profiling

A. Metastasising OMMs

| **Dog ID.** | **Age of FFPE tumour at RNA isolation** (Years) | **RNA integrity**  (SINE Cq) |
| --- | --- | --- |
| D1 | 7 | 26.98 |
| LR1 | 13 | 31.92 |
| CB1 | 15 | 33.15 |
| CB2 | 17 | 32.39 |
| LR2 | 8 | 31.50 |
| D2 | 10 | 29.56 |
| LR3 | 10 | 28.82 |
| GR1 | 6 | 24.97 |
| CS1 | 6 | 25.49 |
| GR2 | 4 | 23.40 |
| CB3 | 4 | 30.96 |
| CB4 | 5 | 28.32 |
| CB5 | 4 | 30.79 |
| GR3 | 3 | 31.98 |
| GR4 | 4 | 25.43 |
| BM1 | 4 | 22.74 |
| GS1* | 4 | 30.08 |
| CB6 | 5 | 29.31 |
| SB1* | 4 | 22.49 |
| BC1 | 7 | 23.89 |

*'Outlier RNA samples' excluded prior to differential gene expression analysis

Prior to ‘outlier array’ exclusion

| Mean and standard deviation | 7.00 ± 3.92 | 28.21 ± 3.42 |
| --- | --- | --- |
| Median | 5.50 | 29.07 |
| Interquartile range | 4.50 | 5.78 |

Following ‘outlier array’ exclusion

| Mean and standard deviation | 7.33 ± 4.00 | 28.42 ± 3.30 |
| --- | --- | --- |
| Median | 6.00 | 29.07 |
| Interquartile range | 5.50 | 5.92 |

B. Non-metastasising OMMs

| **Dog ID.** | **Age of FFPE tumour at RNA isolation** (Years) | **RNA integrity** (SINE Cq) |
| --- | --- | --- |
| GD1 | 19 | 25.03 |
| LR6* | 17 | 33.49 |
| GR5 | 11 | 23.32 |
| GR6 | 19 | 21.66 |
| LR4 | 12 | 30.20 |
| A1* | 12 | 22.16 |
| LR5 | 16 | 26.27 |
| GR7 | 10 | 30.75 |
| IT1 | 9 | 30.46 |
| BF1 | 6 | 33.24 |
| GR8 | 7 | 31.04 |
| CB7 | 7 | 31.79 |

*'Outlier RNA samples' excluded prior to differential gene expression analysis

Prior to ‘outlier array’ exclusion

| Mean and standard deviation | 12.08 ± 4.46 | 28.28 ± 4.15 |
| --- | --- | --- |
| Median | 11.50 | 30.33 |
| Interquartile range | 7.75 | 6.63 |

Following ‘outlier array’ exclusion

| Mean and standard deviation | 11.60 ± 4.61 | 28.38 ± 3.77 |
| --- | --- | --- |
| Median | 10.50 | 30.33 |
| Interquartile range | 7.50 | 5.63 |

Table S3. RNA sample quality metrics for identification of ‘outlier’ arrays

| **Dog ID.** | **pm_mean** | **bgrd_mean** | **all_probeset_mean** | **all_probeset_rle_mean** | **all_probeset_mad_residual_mean** | **pos_vs_neg_auc** | **%P** |
| --- | --- | --- | --- | --- | --- | --- | --- |
| D1 | 56.04 | 38.46 | 2.45 | 0.68 | 0.33 | 0.67 | 14.30 |
| LR1 | 44.22 | 38.86 | 2.34 | 0.66 | 0.37 | 0.62 | 6.84 |
| CB1 | 61.76 | 51.80 | 2.33 | 0.65 | 0.35 | 0.60 | 8.92 |
| CB2 | 45.26 | 37.25 | 2.34 | 0.66 | 0.36 | 0.62 | 7.82 |
| LR2 | 68.09 | 56.02 | 2.35 | 0.64 | 0.34 | 0.65 | 8.50 |
| D2 | 64.35 | 47.03 | 2.40 | 0.60 | 0.32 | 0.67 | 12.37 |
| LR3 | 60.50 | 51.08 | 2.36 | 0.61 | 0.32 | 0.66 | 8.25 |
| GR1 | 95.45 | 48.89 | 2.49 | 0.75 | 0.30 | 0.73 | 22.17 |
| CS1 | 67.80 | 44.41 | 2.45 | 0.62 | 0.30 | 0.70 | 16.35 |
| GR2 | 76.99 | 40.35 | 2.55 | 0.79 | 0.31 | 0.74 | 24.08 |
| CB3 | 43.18 | 43.01 | 2.30 | 0.75 | 0.40 | 0.59 | 4.15 |
| CB4 | 53.76 | 48.87 | 2.32 | 0.65 | 0.35 | 0.65 | 5.12 |
| CB5 | 60.24 | 59.62 | 2.31 | 0.72 | 0.36 | 0.61 | 5.01 |
| GR3 | 63.11 | 46.68 | 2.32 | 0.67 | 0.35 | 0.65 | 7.57 |
| GR4 | 69.02 | 39.28 | 2.51 | 0.72 | 0.31 | 0.73 | 20.86 |
| BM1 | 82.37 | 43.27 | 2.53 | 0.77 | 0.30 | 0.75 | 23.75 |
| GS1^a^ | 77.57 | 69.08^b^ | 2.32 | 0.61 | 0.31 | 0.62 | 7.73 |
| CB6 | 51.23 | 54.95 | 2.30 | 0.79 | 0.39 | 0.56 | 3.70 |
| SB1^a^ | 110.16^b^ | 49.89 | 2.61^b^ | 0.90^b^ | 0.30 | 0.76 | 33.20^b^ |
| BC1 | 75.73 | 53.17 | 2.44 | 0.58 | 0.27 | 0.70 | 16.99 |
| GD1 | 55.47 | 42.78 | 2.42 | 0.59 | 0.30 | 0.68 | 12.41 |
| LR6^a^ | 62.58 | 58.23 | 2.30 | 0.80 | 0.41^b^ | 0.59 | 4.64 |
| GR5 | 90.16 | 51.30 | 2.51 | 0.69 | 0.28 | 0.71 | 25.33 |
| GR6 | 86.25 | 44.82 | 2.53 | 0.78 | 0.31 | 0.72 | 28.73 |
| LR4 | 68.73 | 48.94 | 2.32 | 0.70 | 0.36 | 0.60 | 8.36 |
| A1^a^ | 91.96 | 46.50 | 2.59^b^ | 0.86^b^ | 0.31 | 0.75 | 31.67^b^ |
| LR5 | 68.82 | 54.19 | 2.34 | 0.60 | 0.30 | 0.63 | 10.48 |
| GR7 | 61.65 | 50.51 | 2.36 | 0.60 | 0.32 | 0.65 | 9.13 |
| IT1 | 52.36 | 42.75 | 2.34 | 0.62 | 0.34 | 0.64 | 8.88 |
| BF1 | 53.21 | 47.37 | 2.31 | 0.68 | 0.36 | 0.64 | 5.60 |
| GR8 | 55.75 | 48.89 | 2.37 | 0.62 | 0.35 | 0.66 | 7.83 |
| CB7 | 38.47 | 38.98 | 2.31 | 0.71 | 0.39 | 0.61 | 4.24 |

^a^Outlier arrays and ^b^associated RNA sample quality metric^3,4^

Table S4. Reagents used in quantitative PCR assays

A. RNA integrity assay

| **Gene ID.** | **Forward primer sequence**  (5'-3') | **Reverse primer sequence**  (5'-3') | **PCR product size** (bp) |
| --- | --- | --- | --- |
| CfSINE126 | GATCCCTGGGTGGCGCA | GAGACACAGGCAGAGGGAGA | 126 |

B. Assay for reverse transcription and/or PCR inhibitors

| **ID.** | **Sequence** (5'-3') |
| --- | --- |
| SPUD Amplicon | AACTTGGCTTTAATGGACCTCCAATTTTGAGTGTGCACAAGCTATGGAACACCACGTAAGACATAAAACGGCCACATATGGTG  CCATGTAAGGATGAATGT |
| SPUD Forward Primer | AACTTGGCTTTAATGGACCTCCA |
| SPUD Reverse Primer | ACATTCATCCTTACATGGCACCA |

C. RT-qPCR assays for quantitation of gene expression (optimal probe and primer concentrations, established experimentally, are listed)

*TaqMan assays*

|  | **Probe** |  | **Forward Primer** |  | **Reverse Primer** |  |  |  |
| --- | --- | --- | --- | --- | --- | --- | --- | --- |
| **Gene ID.** | **Sequence** (5'-3') | **Conc.** (nM) | **Sequence** (5'-3') | **Conc.** (nM) | **Sequence** (5'-3') | **Conc.** (nM) | **PCR product size** (bp) | **Reaction efficiency** (%) |
| *SNORA61* | AGCACTGCAGGGCCAGATTCAC | 150 | GGTCTTGGTGGTCGTAAA | 900 | GCCCACAAGCTATATACCA | 900 | 82 | 104.98 |
| *SNORD104* | CCGCGTCAGCAGTCTAACAC | 150 | CAGTGATGACATTCCAGA | 900 | GACTGTAGTTCGCATCA | 300 | 65 | 86.04 |
| *DPM2* | CATCCACAAGTACTTCCTGCCCAG | 250 | CCTTCATCGACAGTCAGC | 300 | GATGGCGACAGCATAGG | 300 | 65 | 103.58 |
| *SLC25A51* | CTGTACGAGGACTTATCTTGCCTTCTC | 150 | TGCAGAAGACAACTACAC | 900 | GGGTACTGATATGCTTCC | 900 | 81 | 96.10 |
| *RPL29* | CCTGGGCTTCTGTCTGCATG | 250 | GGAAGGACTGGTATGAC | 300 | CCGCAGGTTTATTTGTAC | 900 | 81 | 89.20 |
| *SNORA76* | CATAACTGGGGCCACCTGGT | 150 | TGTCCTGTGACTTTGAG | 300 | CTTGCGGTTAAAAGAGTC | 900 | 84 | 82.37 |
| *ADAM10* | CTCTCTGCCCAGCCTCTGATCCT | 250 | GAAGGAATATGTAATGGCATCA | 300 | TGCCGATTACAGTCTGTA | 900 | 77 | 114.68 |

*SYBR Green assays*

|  | **Forward Primer** |  | **Reverse Primer** |  |  |  |
| --- | --- | --- | --- | --- | --- | --- |
| **Gene ID.** | **Sequence** (5'-3') | **Conc.** (nM) | **Sequence** (5'-3') | **Conc.** (nM) | **PCR product size** (bp) | **Reaction efficiency** (%) |
| *PQLC1* | TGCAGATAATAAGGACGAAGAAGT | 800 | CAGATACAACCGCCTGGG | 300 | 52 | 114.46 |
| *CXCL12* | ACCTGCATTTATAGTGTCTGGT | 800 | GGCACCGGTGAATATAAGCTG | 300 | 52 | 108.54 |
| *RBM3* | TCTGCTTCTCGGCGGTT | 500 | CTGCAAATCAAACAAAAAGCTCAAT | 300 | 50 | 91.08 |
| *SNORD61* | AGCTGTGATGTATTTAATTG | 500 | AGCTCAGAACTTCTTAGA | 800 | 72 | NM |
| *APOBEC3A* | TGATTTTCGATGCAGAGC | 500 | TTGGTAGAAGATCCTTAATATTTCC | 300 | 50 | 85.46 |
| *CfSINE71^a^* | GGAGACCCGGGATCGAAT | 300 | GAGACACAGGCAGAGGGAG | 300 | 71 | 94.66 |

^a^Reference gene for calculation of relative gene expression levels

NM - The reaction efficiency could not be calculated because amplification of the target mRNA could only be detected when assaying a preamplified cDNA template prepared from 2µg of a pool of OMM total RNAs.

Table S5. Assay for reverse transcription and PCR inhibitors in OMM RNAs

SPUD amplicon alone (*in the presence of reverse transcription buffer: 1.5µl of ‘-RNA’ reverse transcription reaction product in a 10µl PCR reaction*) Cq = 23.50 ± 0.03

A. Metastasising OMMs

| **Dog ID.** | **Mean Cq** | **Cq SD** | **Cq - SPUD Amplicon Cq** |
| --- | --- | --- | --- |
| D1 | 23.89 | 0.07 | 0.39 |
| LR1 | 23.74 | 0.03 | 0.24 |
| CB1 | 23.74 | 0.06 | 0.24 |
| CB2 | 23.65 | 0.02 | 0.15 |
| LR2 | 23.88 | 0.09 | 0.38 |
| D2 | 23.75 | 0.12 | 0.25 |
| LR3 | 23.60 | 0.00 | 0.10 |
| GR1 | 23.68 | 0.04 | 0.18 |
| CS1 | 23.82 | 0.05 | 0.32 |
| GR2 | 23.74 | 0.14 | 0.24 |
| CB3 | 23.60 | 0.03 | 0.10 |
| CB4 | 23.63 | 0.02 | 0.13 |
| CB5 | 23.77 | 0.05 | 0.27 |
| GR3 | 23.80 | 0.10 | 0.30 |
| GR4 | 23.64 | 0.06 | 0.14 |
| BM1 | 23.64 | 0.02 | 0.14 |
| CB6 | 23.74 | 0.08 | 0.24 |
| BC1 | 23.70 | 0.08 | 0.20 |
|  |  |  |  |
| Mean and standard deviation | 23.72 ± 0.09 |  | 0.22 ± 0.09 |
| Median | 23.74 |  | 0.24 |
| Interquartile range | 0.12 |  |  |

B. Non-metastasising OMMs

| **Dog ID.** | **Mean Cq** | **Cq SD** | **Cq - SPUD Amplicon Cq** |
| --- | --- | --- | --- |
| GD1 | 23.58 | 0.04 | 0.08 |
| GR5 | 23.65 | 0.06 | 0.15 |
| GR6 | 23.82 | 0.02 | 0.32 |
| LR4 | 23.74 | 0.06 | 0.24 |
| LR5 | 23.77 | 0.05 | 0.27 |
| GR7 | 23.65 | 0.07 | 0.15 |
| IT1 | 23.77 | 0.10 | 0.27 |
| BF1 | 23.65 | 0.06 | 0.15 |
| GR8 | 23.52 | 0.09 | 0.02 |
| CB7 | 23.69 | 0.11 | 0.19 |
|  |  |  |  |
| Mean and standard deviation | 23.68 ± 0.09 |  | 0.18 ± 0.09 |
| Median | 23.67 |  | 0.17 |
| Interquartile range | 0.11 |  |  |

**Figures**

Figure S1. Unsupervised hierarchical clustering of 28 OMMs on the basis of the expression values

of the 20% of Transcript clusters (2,648) with the highest variance in expression signal

A. OMMs labelled according to breed B. OMMs labelled according to sex


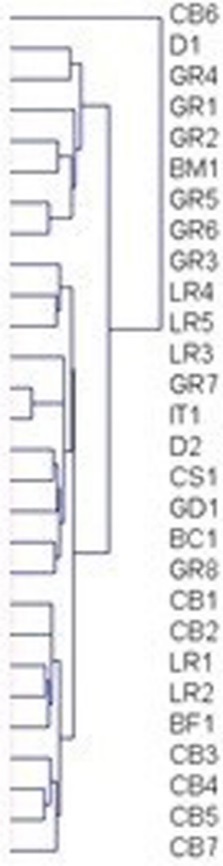


BC - Border Collie, BF - Bouvier des Flandres,

BM - Bullmastiff, CB - Cross breed,

CS - Cocker Spaniel, D - Dachshund,

GD - Great Dane, GR - Golden Retriever,

IT - Irish Terrier, LR - Labrador Retriever


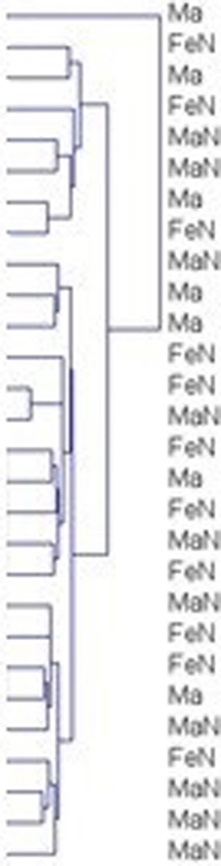


Fe - Female, FeN - Neutered female,

Ma - Male, MaN - Neutered male

C. OMMs labelled according to age


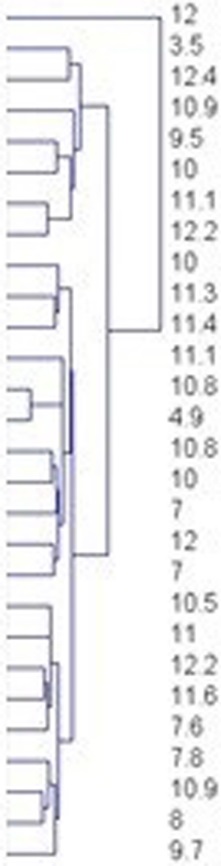


Numbers indicate age in years
